# Data-driven pipeline enables discovery of plant protein surfactants

**DOI:** 10.1038/s42004-026-02173-6

**Published:** 2026-09-03

**Authors:** Simha Sridharan, Rammile Ettelaie, Rik Sarkar, Maryam Afzali Haji Dela, Brent S. Murray, Thomas A. Hazlehurst, Nicholas J. Watson, Anwesha Sarkar

**Affiliations:** 1 Food Colloids and Bioprocessing Group, School of Food Science and Nutrition, University of Leeds, Leeds, UK; 2 School of Informatics, University of Edinburgh, Edinburgh, UK; 3 National Alternative Protein Innovation Centre (NAPIC), Leeds, UK

**Keywords:** Proteins, Statistical mechanics, Polymer chemistry

## Abstract

Designing amphiphilic block copolymers from fossil fuel-derived hydrocarbons is a cornerstone of colloid chemistry, enabling the creation of advanced polymeric surfactants. Despite the high attractiveness of low carbon-emitting plant proteins to defossilise surfactant processing, attempts in identifying plant proteins that will function as appropriate surfactants are somewhat hit and miss. Here, we present a data-driven pipeline to fingerprint plant protein surfactants by harnessing the diblock-like signature of a classic surfactant, integrating protein sequence data with statistical thermodynamics-based calculations, finetuned by machine learning. We demonstrate that protein sequence-level features do not in themselves allow for the prediction of adsorbed configuration. Instead, segmenting adsorbed proteins into blocks enabled identification of hundreds of plant proteins possessing a diblock-like signature, validated experimentally for optimal surface properties. Thus, by embedding adsorption configuration information into the classification process, we streamline the discovery of plant protein surfactants at scale, unlikely to be realised by purely empirical screening.

## Abstract (web summary)

Amphiphilic block copolymers from fossil fuels are an important source of polymeric surfactants, but identifying plant proteins as sustainable surfactants remains challenging. Here, the authors develop a data-driven pipeline combining protein sequence data, statistical thermodynamics, and machine learning to identify plant proteins with optimal surfactant properties.

## Introduction

Synthesis of amphiphilic diblock copolymers with the presence of two distinct hydrophilic and hydrophobic parts has resulted in some of the most pervasive polymeric surfactants for stabilisation of emulsions in technological applications^1–5^. Such polymeric surfactant synthesis has relied on reaction chemistry of monomers derived primarily from fossil carbon sources, resulting in various environmental consequences^6,7^. One of the challenges at the forefront of colloid chemistry today is to relinquish our reliance on fossil fuel-derived hydrocarbon-based polymeric surfactants and transition towards more sustainable polymers. Plant proteins with their complex distribution of hydrophilic, charged, and hydrophobic amino acids (AA) along their polypeptide backbone undoubtedly stand out as a novel class of naturally occurring polymeric surfactants with a low anthropogenic carbon footprint^8^. However, the discovery of plant proteins with targeted surfactant properties remains a formidable challenge. This difficulty stems from navigating through a vast search space of tens of millions of AA sequences of plant proteins in protein data bank (UniProt)^9^, making exhaustive experimental screening unrealistic, even with the support of computational simulations. Despite a surge of successful empirical evidence of specific plant proteins acting as emulsifiers over the last few decades^10–13^, two fundamental questions persist: (1) can plant proteins adopt a specific configuration upon adsorption resembling those of polymeric diblock surfactants? (2) How to systematically choose plant proteins that can function as an optimal surfactant to stabilise emulsions?

Adsorption is the first and the most decisive step in emulsification *via* any polymeric surfactant, where it facilitates the stabilisation of liquid-liquid dispersions such as oil-in-water (O/W) and water-in-oil (W/O) emulsions. Adsorption involves a delicate, dynamic balance between the polymer’s intrinsic properties such as molecular size, hydrophobicity, hydrophilicity and charge distributions and thermodynamic driving forces dictating preference for interface over the bulk solution^14^. Amphiphilic diblock copolymers are highly effective surfactants because they contain distinct hydrophobic domains that anchor onto hydrophobic surface whilst the hydrophilic blocks extend away into the solvent phase, often attaining a classic *S-shaped* configuration among others^15^. Proteins often form such diblock-like configurations, governed by their adsorption energies, specifically, the balance of hydrophobic, electrostatic, and van der Waals interactions^16,17^. The structural rigidity of the adsorbed protein film, charge distribution and strength of anchoring of blocks of AAs to the hydrophobic surfaces significantly impact the rate of protein adsorption and stabilisation of emulsions^18–21^. Experimental approaches, such as tensiometry, scattering, microscopy although vital for analysing protein adsorption often lack the molecular resolution needed to test protein adsorption and identify proteins that will certainly form stable, functional films at oil-water interfaces^22,23^. Of more importance, such analyses can take an exceptionally long time from days or even weeks to determine equilibrium properties of adsorbed proteins, further complicating identification of protein surfactants from a choice of millions of plant proteins.

Lattice self-consistent field theory (SCFT) implemented via the Scheutjens–Fleer approach offers a robust, computationally efficient, mean-field, statistical mechanical technique, to predict equilibrium configurations and densities^24^ and conformational entropy of plant proteins at the surface^24–26^. Despite being a powerful, scalable method, SCFT has never been attempted at scale for protein adsorption configuration. The advent of data science and large protein databases in the last few years has revolutionised the scale of quantitative understanding of protein structure-function relationships^27,28^. Nevertheless, state-of-the-art deep learning-based structure prediction models such as AlphaFold3 and protein language models such as Evolutionary Scale Modelling 2 (ESM-2) are primarily designed and optimised to predict the native, stable, three-dimensional structure of a protein in an aqueous environment. Consequently, they face significant limitations in capturing the physical aspects of proteins interacting with surfaces^29,30^, where proteins denature and become disordered upon adsorption. Specifically, these models do not incorporate surface interaction parameters, even on a rather simplified level (e.g., the Flory-Huggins type (*χ*) parameters)^31^. Therefore, an adsorption configuration-informed machine learning approach is a necessary logical undertaking^32^ for the rational selection of plant protein surfactants, which has so far been completely overlooked^33,34^ in the literature.

Herein, we describe a data-driven, mechanistic, discovery pipeline to identify plant proteins that will adopt a diblock-like configuration upon adsorption and therefore, on this level, behave as an optimal polymeric surfactant. By integrating SCFT calculations and machine learning (ML), our high-throughput computational pipeline systematically interrogates for the protein to have a signature for diblock-like adsorption configuration on an unprecedented scale. We define diblock score as a novel mathematical measure for statistically quantifying the diblock-like nature of plant proteins. We demonstrate that ML-based classification using block-level AA features with diblock score of ~0.5 enables to identify plant proteins with diblock-like architecture, resembling that of *β*-casein – an intrinsically disordered protein in nature that achieves a near-ideal diblock-like configuration, thus mimicking classic synthetic polymeric surfactants^18,35,36^. We evaluate our hypothesis experimentally using a subset of these identified plant proteins behaving as optimal surfactants for oil-in-water emulsions. Thus, our study establishes a unique, scalable, resource-efficient discovery pipeline for high-throughput screening to guide the selection of surfactants from the plant kingdom, focused on an unexplored adsorption configuration-based classification.

## Results and Discussion

### Discovery pipeline for diblock-like proteins behaving as optimal surfactants

**Fig. 1:**
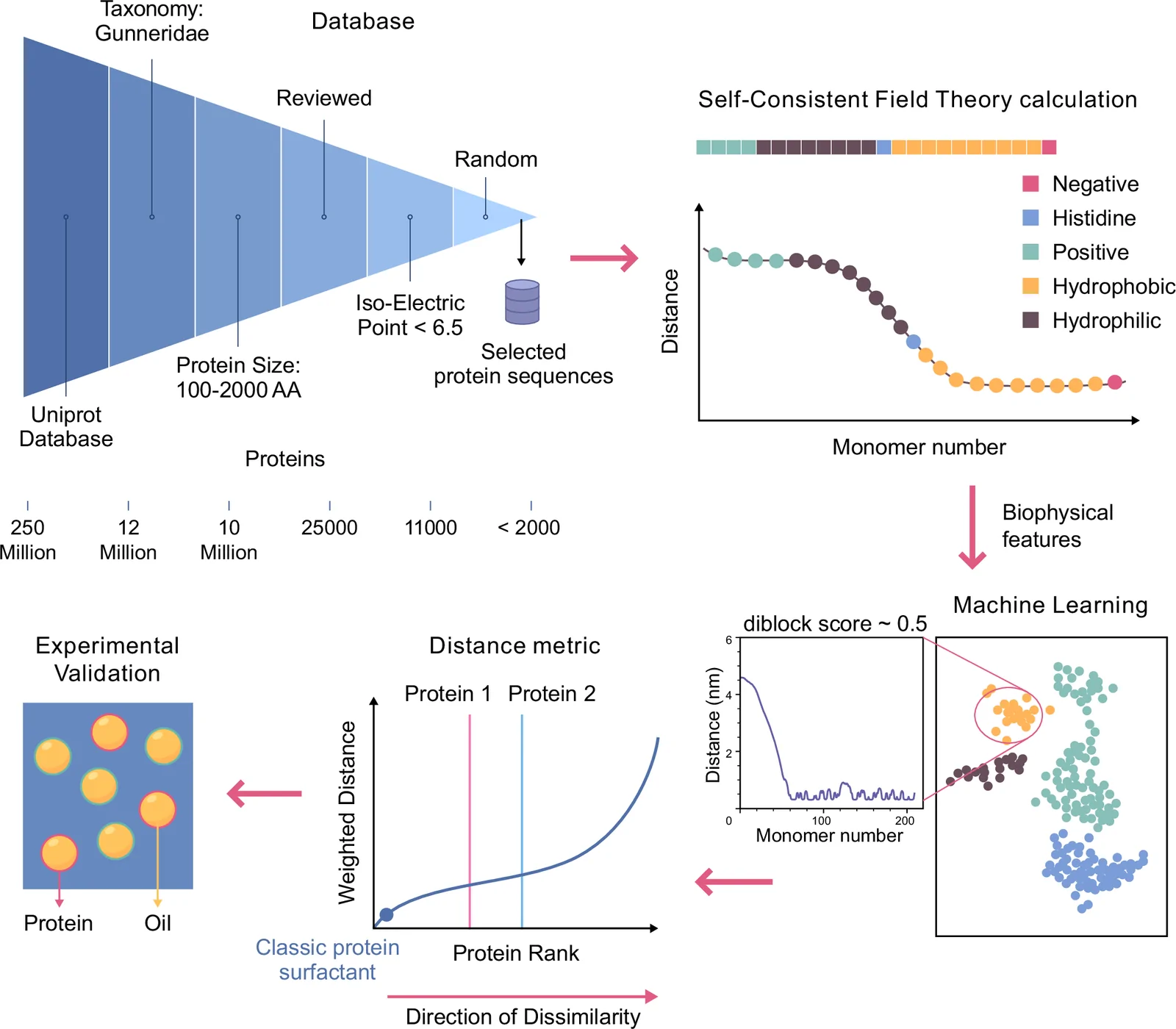
Data-driven discovery pipeline for fingerprinting plant proteins behaving as optimal surfactants. A plant protein database was curated from Protein Data Bank (PDB) of 250 million proteins to a set of 1950 plant proteins with an IEP below pH 6.5 and amino acid (AA) length between 100 and 2000. Self-consistent field theory (SCFT) calculations were applied at pH 7.0 and salt concentration of 1 mM NaCl. Proteins which exhibited diblock-like adsorption configuration was clustered and block-level feature importance was evaluated using machine learning (ML) and used to build a distance metric for diblock-like proteins. Our hypothesis was experimentally tested on a subset of chemically disordered plant proteins (closest distance to classic protein surfactant) using biophysical characterisation (surface tension, droplet sizing, confocal laser scanning microscopy).

To develop the data-driven pipeline to identify plant proteins behaving as optimal surfactant, we combined the protein sequence data from the UniProt repository (https://www.uniprot.org/)^9^ with physical principles governing adsorption to hydrophobic surfaces, as reflected in SCFT calculations, followed by a completely unexploited block-level protein analysis empowered by ML. We developed a five-step process (Fig. 1) to select 1950 experimentally reviewed plant protein sequences from a vast 250 million proteins. First, we selected taxonomy *Gunneridae*, which represente land-based plants. Further, we screened these proteins based on size (100–2000 AA), iso-electric point (IEP) below pH 6.5. Isoelectric point (IEP) of 6.5 was chosen as many, if not, most commercially applied diblock surfactants for industrial applications carry a negative charge at neutral pH. Since our reference protein was *β*-casein, which also carries a negative charge at neutral pH, it intuitive to narrow down the screening to negatively charged proteins. While positively charged proteins may also be excellent diblock-like surfactants, they were excluded in this work to build the subset for SCFT simulation that represents a reasonably large section of industrially relevant surfactants that are prevalent in chemical applications. Although, the SCFT and ML findings to build this discovery pipeline here are limited to these proteins, our methodology can be broadly applied to positively charged proteins in the future. We selected the cut-off of 100–2000 AAs to balance a broad range of commonly used proteins and computation times needed in SCFT simulations.

The SCFT computations yielded protein adsorption configurations for 1,858 of these plant proteins (95% convergence success) and showed various types of adsorption configurations such as diblock-like (e.g., proteins including Scarecrow like protein 15 Arabidopsis, length 486 AAs, which is a metabolic regulatory protein found in plants), single loops (e.g., Alpha amylase 1 Arabidopsis thaliana, length 399 AAs), or multi-loop-like configurations (e.g., gamma cadinene synthase Cucumis melo, length 571 AAs) (see Suppl. Fig. 1). The 5% unconverged proteins (92 proteins) do not represent a unique class of proteins (Suppl. Fig. 2), rather they represent proteins that were difficult to converge representing a numerical limit of the subroutine solvers in our computational implementation of SCFT.

We restrained clustering analysis *via* physical feature extraction from the SCFT-derived adsorbed configurations. To identify an optimal surfactant, a diblock like adsorption configuration was chosen since it represents the simplest adsorption configuration and is a well-known configuration adopted by proteins such as *β*-casein, which has been validated as an excellent emulsifier experimentally and theoretically over several decades^18,35,36^.

We defined a statistical diblock score which enabled us to identify diblock-like plant protein clusters with confidence (Fig. 1). To benchmark diblock-like signature of proteins against a well-known diblock protein emulsifier, we compared the adsorption curves of tested proteins to that of a classic model protein surfactant with diblock like configuration i.e., *β*-casein (Suppl. Fig. 3). Decades of research has confirmed that this diblock-like structure with distinct polar/charged and hydrophobic blocks^15,18,35,37^ is a key contributor to exceptional stabilizing performance of *β*-casein in emulsions, akin to synthetic polymeric surfactants. To identify features similar to *β*-casein, we analyse the attached and the free blocks of the diblock-like plant proteins, separately. We quantified the relative importance of features per block such as, polar fraction, hydrophilic fraction, charged residues fraction, etc. by measuring their contribution in a Random Forest classifier. These feature importances were used to construct a 14-dimensional Euclidean space to rank the diblock-like plant proteins by their similarity to *β*-casein. Experiments show that a subset of plant proteins predicted as having a diblock-like adsorption configuration, indeed stabilise oil-in-water emulsions, supporting the robustness of this pipeline and the hypothesis to navigate the vast plant protein space and identify previously unanticipated plant protein-based surfactants (Fig. 1). It is essential to note that while *β*-casein represents intrinsically disordered protein, many plant proteins have complex 3D structures, which SCFT model does not take into consideration. Notably, SCFT is probing interface behaviour where many, if not, most plant proteins can partially unfold and can behave less rigid than in solution. Furthermore, in industrially relevant thermal processing conditions, often proteins are more flexible owing to thermal denaturation. Nevertheless, future work needs to account for 3D structure of proteins built into the model to understand the importance of partial unfolding on adsorption.

### Protein sequence-level features do not predict diblock-like configuration

**Fig. 2:**
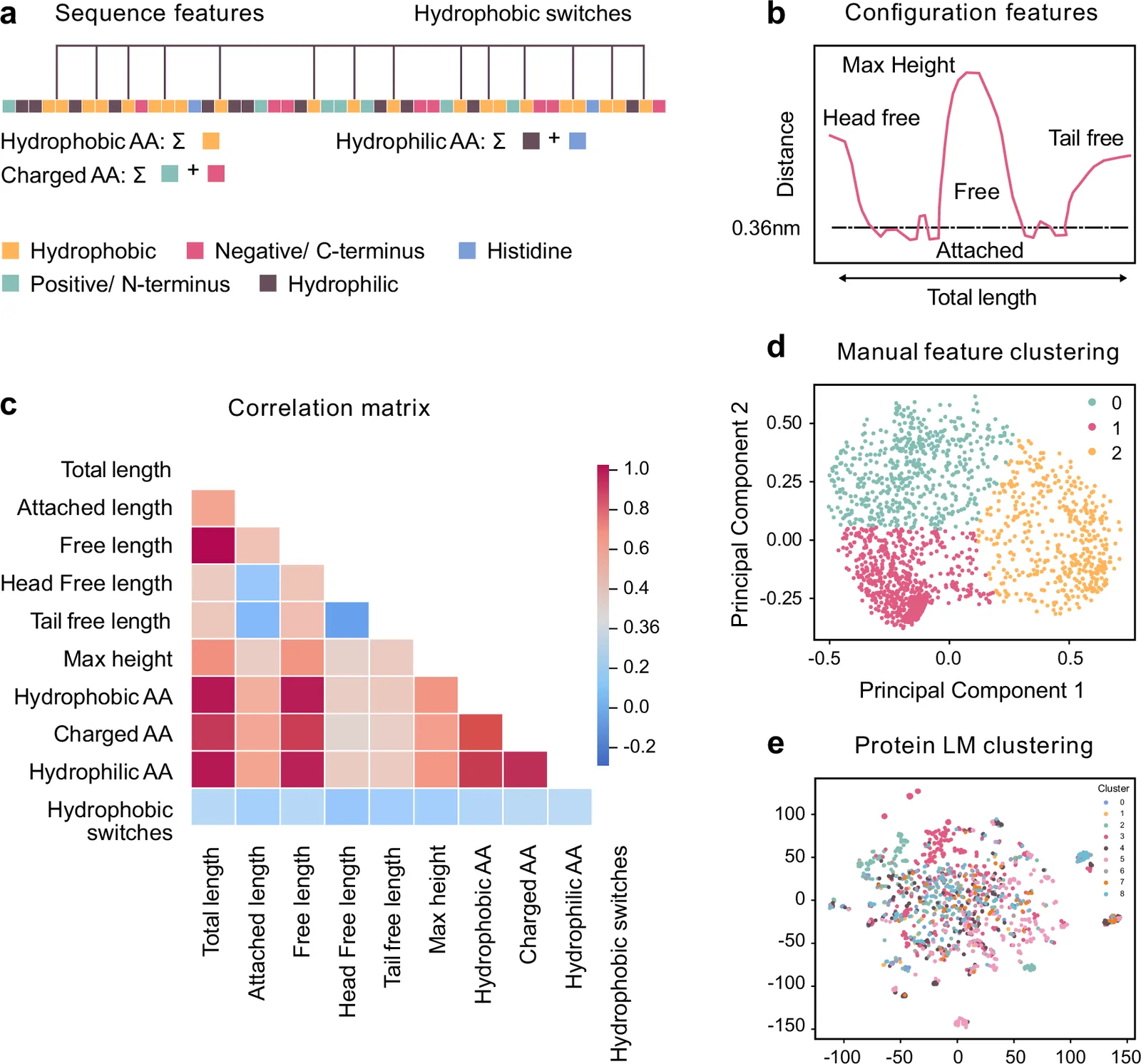
Correlation of protein sequence-level features to adsorption configuration in plant proteins. **a** Illustration showing different sequence-level features of the entire protein extracted for correlation analysis. The features include counts of hydrophilic AAs, hydrophobic AAs and charged AAs, as well as distributional features such as number of hydrophobic switches, which counts the number of hydrophobic-non-hydrophobic switches along the polypeptide chain (see the illustration). **b** Illustration showing extraction of configuration features such as Head free (N-terminal > 0.36 nm), Tail free (C-terminal > 0.36 nm), Total free (number of AA > 0.36 nm), Total attached (number of AA < 0.36 nm), Max height (maximum extension away from the surface) and total length extracted from 1858 SCFT adsorption configuration graphs. **c** Correlation matrix of protein sequence features and adsorption profile features show red as positive correlation and blue for negative correlation and lighter colours for weaker correlation and darker colour for stronger correlation (only the lower triangle is shown since the matrix is diagonally symmetric). **d** *K*-means clustering plot showing clustering of proteins based on the protein sequence features and attached configuration showing poor cluster separation. **e** PCA clustering of proteins using ProtTrans Protein language model embedding, show poor cluster separation unable to identify diblock-like plant protein clusters, visualized using t-stochastic neighbour embedding (t-sne).

Having the selected protein sequences in the discovery pipeline, it is first tempting to ask whether protein sequence-level features can identify diblock-like signatures in plant proteins? In other words, can the number of hydrophobic amino acids (AA), the number of hydrophilic AA and charged AA predict the adsorbed configuration, e.g., what will be the attached length, what will be the non-adsorbed free lengths? To examine this, we extracted sequence-level features from plant proteins such as hydrophobic, hydrophilic, charged AA counts plus atypical features such as ‘hydrophobic switching (transition from a hydrophobic AA to a hydrophilic one as one moves along the backbone, and vs versa)’ from the protein sequences (Fig. 2a). We then extracted adsorption configuration features (Fig. 2b) from the 1858 calculated SCFT curves, such as maximum height, which is the furthest extent of the protrusion of the adsorbed polymer chain away from the surface, total attached length (number of AA at distance < 0.36 nm from the surface) and free length (number of AA at distance > 0.36 nm from the surface).

A weak correlation exists between protein sequence-level features, such as hydrophilic, hydrophobic, charged AA and adsorption configuration features, such as free length, attached length. (Fig. 2c). Whilst a high hydrophobic AA content can aid adsorption to a non-hydrophilic surface^21^, the total magnitude of hydrophobicity alone offers limited information on adsorption configuration and effectiveness for colloidal stability. Our findings critiques the frequent use of established, theoretical protein hydrophobicity scales (e.g., Kyte-Doolittle^38^, Nozaki-Tanford^39^), or experimental protein surface hydrophobicity measurements based on dye binding using 1-anilinonaphthalene-8-sulfonate (ANS)^40^ for identifying plant protein surfactants as these do not consider the configurational changes, ignoring protein structure and hydration environments altogether. Other unconventional protein sequence-level features, such as the number of switches from hydrophobic to non-hydrophobic AAs (Fig. 2c) also did not show a strong correlation with the attached length. These results pinpoint the importance of entropic considerations, resulting from the loss of configurational entropy upon adsorption, as a crucial factor in determining the adsorption behaviour^41,42^. While such entropic term itself depends on the primary sequence of AAs along the protein backbone, this dependence is complex and not easy to characterise purely based on a few structural coarse-grained parameters as mentioned above.

Protein sequence-level features did not produce any clear separation of plant proteins into distinguishable clusters with a low silhouette score of 0.52, and was not capable of predicting diblock-like adsorbed configuration (Fig. 2d). Since protein-level compositional sequence features failed, we set out to investigate if state-of-the-art protein language models (pLMs)^43^ such as ProtTrans can identify diblock-like plant proteins. Notably, failure of pLM (Fig. 2e) to separate proteins into distinguishable clusters suggests that protein sequence-based embeddings do not fully capture the spatially heterogeneous physical constraints^29,30^, such as interaction of proteins with surfaces. Taken together, our findings confirm that conventional protein sequence-level metrics, such as overall hydrophobicity or charge are insufficient to predict complex adsorption behaviours, specifically failing to identify diblock-like configurations in plant proteins. Therefore, to guarantee the identification of plant proteins that will result definitively in a diblock-like configuration upon adsorption and behave as an optimal surfactant, a closer inspection of the constitutive blocks itself is necessary.

### Block level features detect diblock-like architecture

Synthetic block copolymer-based surfactants, often referred to as diblocks are explicitly designed to contain two blocks of distinct monomers, *i.e*., a hydrophobic and a hydrophilic one. In naturally occurring plant proteins, the blocks consist of a more mixed sequence of AA residues with no obvious separation. Yet, natural proteins such as *β*-casein exhibit overall statistical diblock-like adsorption behaviour, with a hydrophilic ‘head’ region (N-terminus) and a hydrophobic ‘tail’ region (C-terminus) (see Suppl. Fig. 3). We define a statistical diblock-score to generalise the diblock-like nature of such mixed AA sequences as follows. A sequence Q can be thought of as the concatenation of two sub-sequences or blocks h and t, written as Q=h⊕t. The diblock score of any such pair $(h,{t})$ is defined as

1 $$D\left(h,t\right)=\left({2}^{2\eta \left(h\right){|h|}}-1\right)\left({2}^{2\gamma \left(t\right){|t|}}-1\right)$$

where, η(h) measures the hydrophilicity of h, and γ(t) measures the hydrophobicity of t, and $\left|h\right|,{|t|}$ are the fractional lengths of the blocks. A protein Q of length n can be segmented into n+1 such possible pairs (allowing zero length subsequences). The diblock score of Q is simply the best (maximum) of the score over all possible segmentations:

2 $$D\left(Q\right)={\max }_{h\oplus t=Q}D\left(h,t\right)$$

A higher D(Q) suggests a more diblock like distribution of AAs. The function has several desirable properties. For $\gamma ,\eta \in [{{\mathrm{0,1}}}]$, $D\left(Q\right)$ is always in the interval $[{{\mathrm{0,1}}}]$, and equals 0 when either h or t is zero length. It is also continuous in $\gamma ,\eta ,\left|h\right|$ and $\left|t\right|,$ and can be computed efficiently in time linear in n. It prefers a balanced split of $(h,{t})$. for example, for $\gamma \left(\cdot \right)=1,\,\eta \left(\cdot \right)=1$, the maximum is realised for $\left|h\right|=\left|t\right|=0.5.$ This corresponds to perfectly symmetric diblock polymers, which are a frequent subject of analytical study^44^ in polymeric surfactants.

The values $\gamma \left(t\right)$ and η(h) can be computed in different ways. For example, the Kyte-Doolittle hydrophobicity scale^38^ can be used to determine the hydrophobicity (and conversely, hydrophilicity) of each AA, which are then averaged over the length of the subsequence to obtain the value. More meaningful scores for natural proteins can be obtained using the data in the SCFT configurations rather than hydrophobic scales because of their limitations. In this SCFT approach, we determine the hydrophilicity or hydrophobicity of an AA within a chain by comparing its distance from the surface with a pair of thresholds and then averaging as described above. This method produces a $D\left(Q\right)$ of 0.48 for *β*-casein. Hence, *β*-casein is statistically diblock-like but not a perfect diblock (i.e., $D\left(Q\right)$ ~ 1) as one can anticipate with chemically synthesized symmetric diblocks. We now describe a way to partition the plant protein set based on their adsorption configuration and then examine their scores.

#### Block-level binary descriptors and classification

**Fig. 3:**
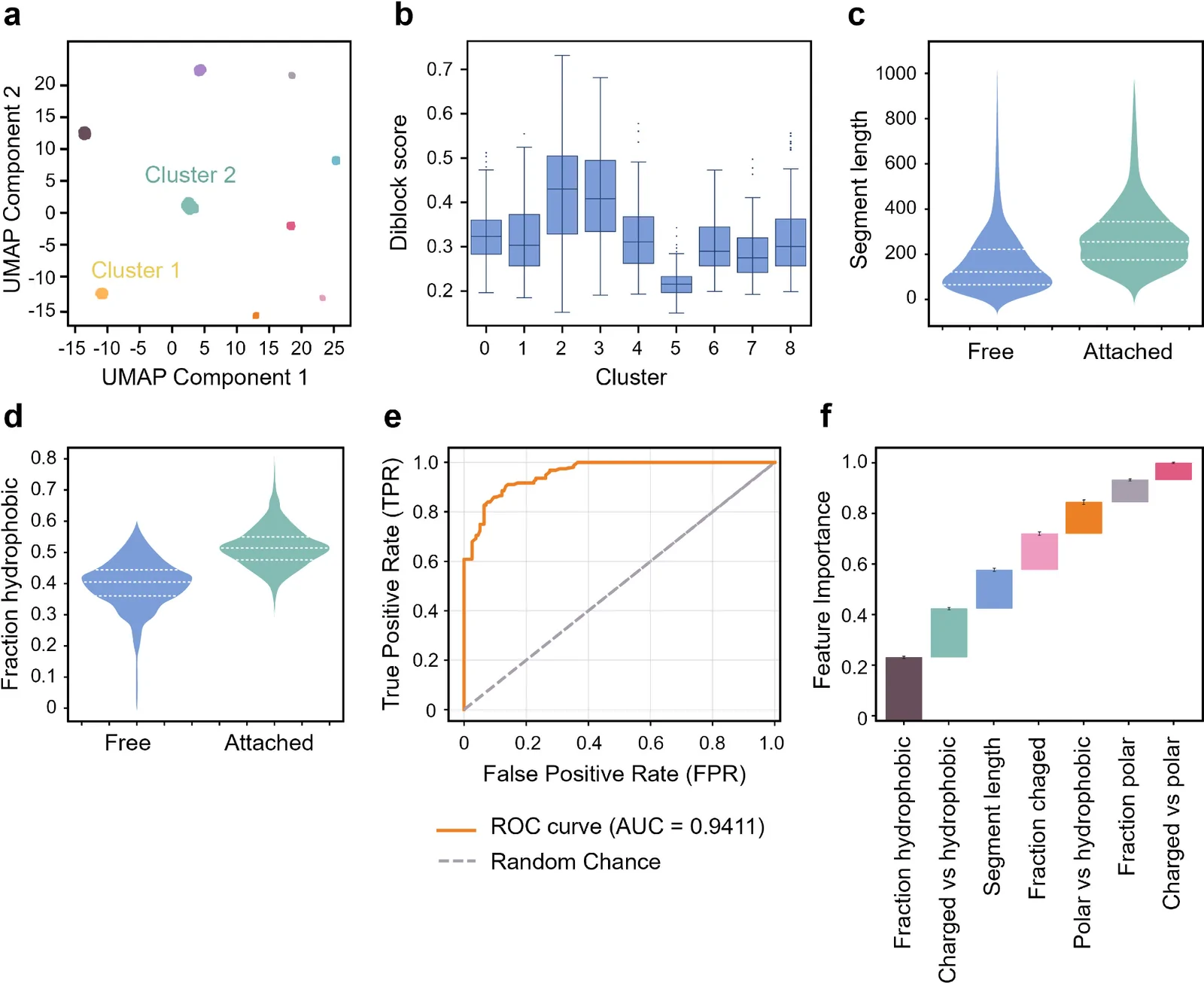
Block-level analysis for identifying signatures of diblock-like plant proteins. **a** Density-based unsupervised clustering (DBSCAN) of 1858 plant proteins based on SCFT-derived adsorption configurations, revealing nine distinct clusters. Clusters 2 (yellow) and 3 (cyan) correspond to diblock-like adsorption configurations. **b** Mean SCFT-based diblock score, *D(Q)*, for each cluster, shown with standard deviation and range; clusters 2 and 3 exhibiting the highest mean *D(Q)*, consistent with diblock-like architecture. **c** Violin plot showing the distribution of block lengths for attached and free blocks across diblock-like proteins. The width of each violin represents the probability density of the data, with the median and interquartile range indicated as dashed lines. **d** Violin plot showing the distribution of hydrophobic amino-acid fraction in attached and free blocks, highlighting systematic compositional differences between the two block types. **e** Receiver operating characteristic (ROC) curve illustrating the ability of block-level features to distinguish attached and free blocks within the SCFT framework using a random forest classifier (Area Under Curve = 0.94); the diagonal dashed line represents random classification. **f** Feature-importance ranking (waterfall plot, which plots from the largest value to smallest value x-category adding up to 1) of seven block-level amino-acid features derived from the random forest analysis. Higher values indicate greater contribution to distinguishing attached from free blocks, rather than predictive power.

The SCFT configurations exhibit natural structures of interest, where some sections of the sequence - the head, tail, or middle is detached and far from the surface. In some cases, a protein may have multiple detached sections. We created a descriptor with four such binary features for each protein (see Suppl. Fig. 4, Suppl. Table 1, see Methods section for details). The plant proteins can be clustered in nine distinct classes according to these descriptors (Fig. 3a) with a high clustering silhouette score of 0.94 (silhouette score measures the similarity of clusters within a cluster label with 1 = perfect clustering and −1 = random clustering). Among them are two large diblock-like clusters corresponding to prominent detached heads and detached tails respectively (see Suppl. Figs. 5 and 6); and in our enumeration these are cluster number 2 and 3. These clusters taken together comprise of 787 plant proteins showing diblock-like configuration in (see exemplars in Supplementary Fig. 7, such as Thaumatin-like protein 1b Fragment OSMalus domestica OX3750 PE2 S, ATP synthase subunit d mitochondrial OSArabidopsis thaliana OX3702). Notably, these clusters 2 and 3 have statistically higher $D\left(Q\right)$ compared to other clusters of proteins, where cluster 2 has a mean $D\left(Q\right)$ of 0.415 and cluster 3 has a mean $D\left(Q\right)$ of 0.402 (Fig. 3b).

#### Block-level analysis of diblock-like plant proteins

Remarkably, the AA sequences in the attached and free blocks of proteins in clusters 2 and 3 show clear patterns. The attached blocks have relatively higher mean hydrophobic AA content of 0.48 fraction, while the free blocks have a lower mean of 0.32 fraction (Fig. 2c, see statistical significance in Suppl. Fig. 8). The attached blocks are also typically longer (median 136 AAs) than the corresponding free blocks (median 73 AAs) (Fig. 3c, d). The free blocks also have higher fraction charged AA (0.29) and higher polar fraction (0.3) compared to attached blocks (0.21 fraction charged and 0.26 fraction polar).

From these discriminative properties, we derived 7 features based on our block-level analysis. We trained a random forest classifier (RFC) to distinguish between attached and free blocks. In our pipeline, RFC is used as an interpretation tool and not as a predictive tool for adsorption behaviour. Therefore, the resulting Area Under Curve (AUC = 0.94) indicate the robustness of the relationship between the features and the adsorbed states. Therefore, feature importance score was computed and displayed in a waterfall plot. A waterfall plot which shows that fraction of hydrophobic AAs is the most discriminative feature at a score of 0.24, followed by ratio of charged and hydrophobic (Fig. 3f, see block-level comparisons between attached and free blocks in Suppl. Fig. 9). We also report comparable AUC and accuracy across multiple classifier models to demonstrate that the observed block-level signal is robust and not dependent on a specific model architecture (Suppl. Fig. 10 and Suppl. Table 2).

#### Comparing diblock proteins and computing similarity to *β*-casein

**Fig. 4:**
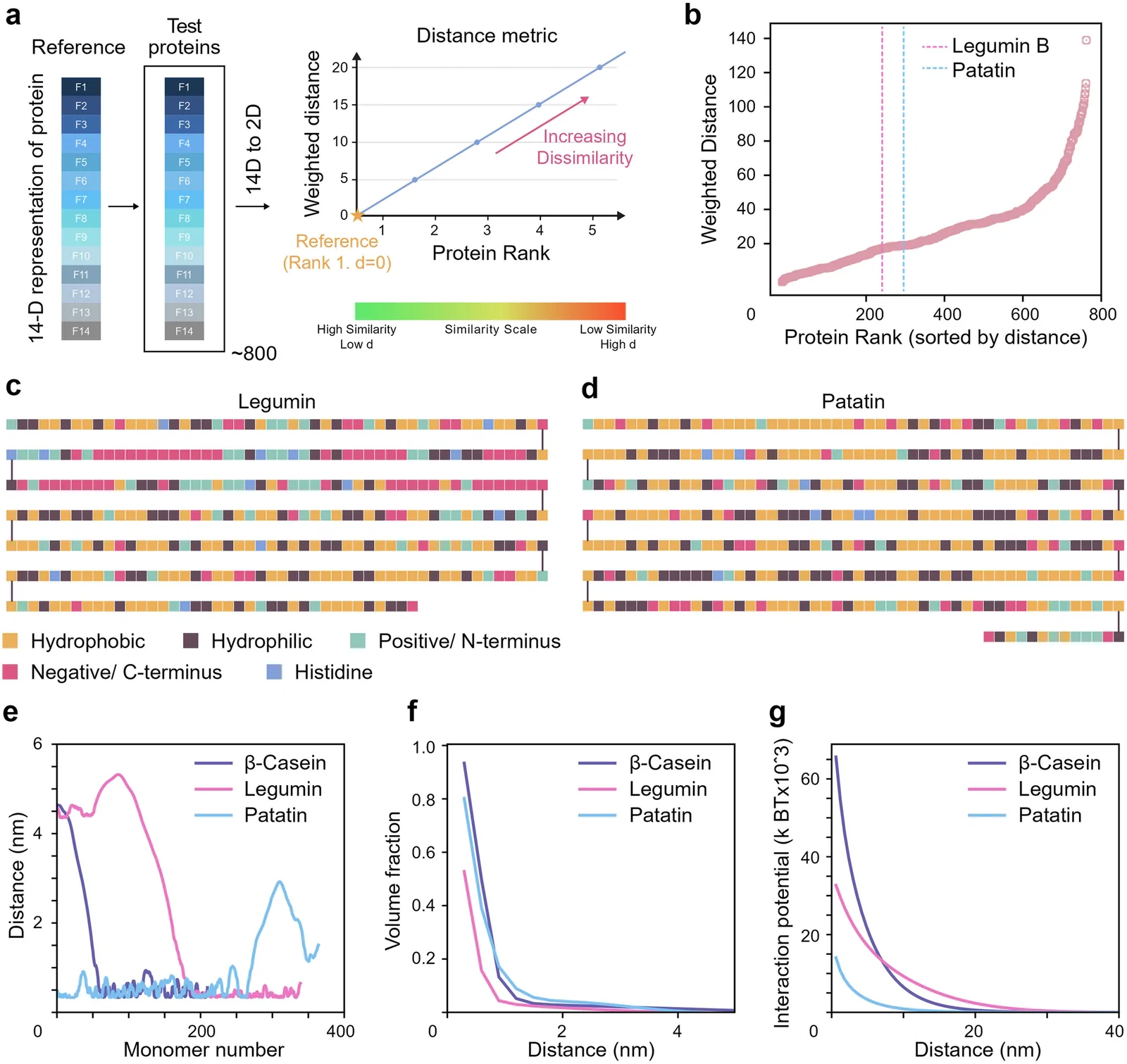
Identifying plant proteins with diblock like adsorption configuration closely resembling that of classic protein surfactant. **a** 14-dimensional weighted feature space for creating distance metric to measure similarity of plant protein-based diblocks to *β*-casein. **b** Euclidean distance of all diblock-like proteins from *β*-casein, where a subset of two proteins, legumin B (extracted from pea) and patatin (extracted from potato) being identified (**c**, **d**) shows the sequence of legumin B and patatin, respectively colour coded for hydrophobic, hydrophilic, positively- and negatively charged as well as Histidine, latter modelled separately**. e** SCFT adsorption configuration of legumin B and patatin in comparison to *β*-casein, used here a benchmark. Both legumin B and patatin show a diblock-like attached configuration in SCFT with some variance from *β*-casein. **f** Volume fraction of legumin B and patatin as compared to *β*-casein, as a function of distance from a hydrophobic surface. **g** Colloidal interaction potentials between two surfaces covered by legumin B and patatin as compared to *β*-casein, plotted as a function of separation distance between two sphere surfaces of diameter 1 µm calculated using the Dergayun’s approximation and represented in *k*_B_T units where *k*_B_ is Boltzmann constant and *T* is absolute temperature.

The 7 features provide a natural space for comparing diblock-like plant proteins. Seven features can be computed for each of the two blocks, resulting in a 14-feature description of diblocks. The distance between any two proteins in this 14-dimensional space is an estimate of the difference in their surfactant-like behaviour within a diblock like adsorption space. In Fig. 4a, we compare and rank diblock-like plant proteins with *β*-casein as the reference. Figure 4b shows the result of ranking plant proteins in the diblock clusters by their distance from *β*-casein, where the distance is measured with coordinates ranked by their feature importance weights.

To test the top-ranked diblock-like proteins identified by the pipeline, experimental validation focused on candidates that are currently accessible at production scale to be testable in experiments, recognising that many of the highest-ranked proteins are not yet available at experimentally testable scale. We therefore selected a subset of diblock plant proteins that were highly ranked and available at scale for use.

First, we chose Pea legumin B (Fig. 4c, e, f). Like *β*-casein, it exhibits a diblock-like configuration (cluster 2, Fig. 3a) with an extended *N*-terminal section and a shorter attached *C*-terminal end section. On the other hand, we chose patatin (Fig. 4d, e, f), which adopts an inverted diblock-like configuration (cluster 3, Fig. 3a). As visually evident from Fig. 4e, pea legumin B has a smaller proportion of its chain lying flat and anchored to the surface as compared to both *β*-casein and patatin. Consequently, the volume fraction profile for legumin B is lower than for both *β*-casein and patatin (Fig. 4f), indicating a weaker propensity to adsorb to the surface^45^. We find that both these plant proteins exhibit lower repulsive potentials at close distances (legumin B: ~35 × 10^3^ *k*_B_T, patatin: ~15 × 10^3^ *k*_B_T) to the surface as compared to *β*-casein (Fig. 4g), which is quantitatively expected by their respective *D(Q)* (Legumin B: 0.69, Patatin: 0.43) and ranking (Fig. 4b). As seen in Fig. 4e, Both *β*-casein and legumin B, on adsorption to the hydrophobic surface, exhibit a more extended N-terminal section (starting at monomer number 1), extending about 5 nm away from the hydrophobic surface, with the rest of the monomers lying relatively flat at the hydrophobic surface. On the other hand, patatin has an extended section at the C-terminal (at the end of the monomer chain) extending about 3 nm from the surface and the rest of the AAs lie relatively close to the surface. Consequently, both *β*-casein and legumin B provide steric forces at longer distances from the surface, resulting in longer-ranged repulsive potentials calculated at pH 7 and 1 mM NaCl concentration.

### Experimental validation stage of the discovery pipeline

**Fig. 5:**
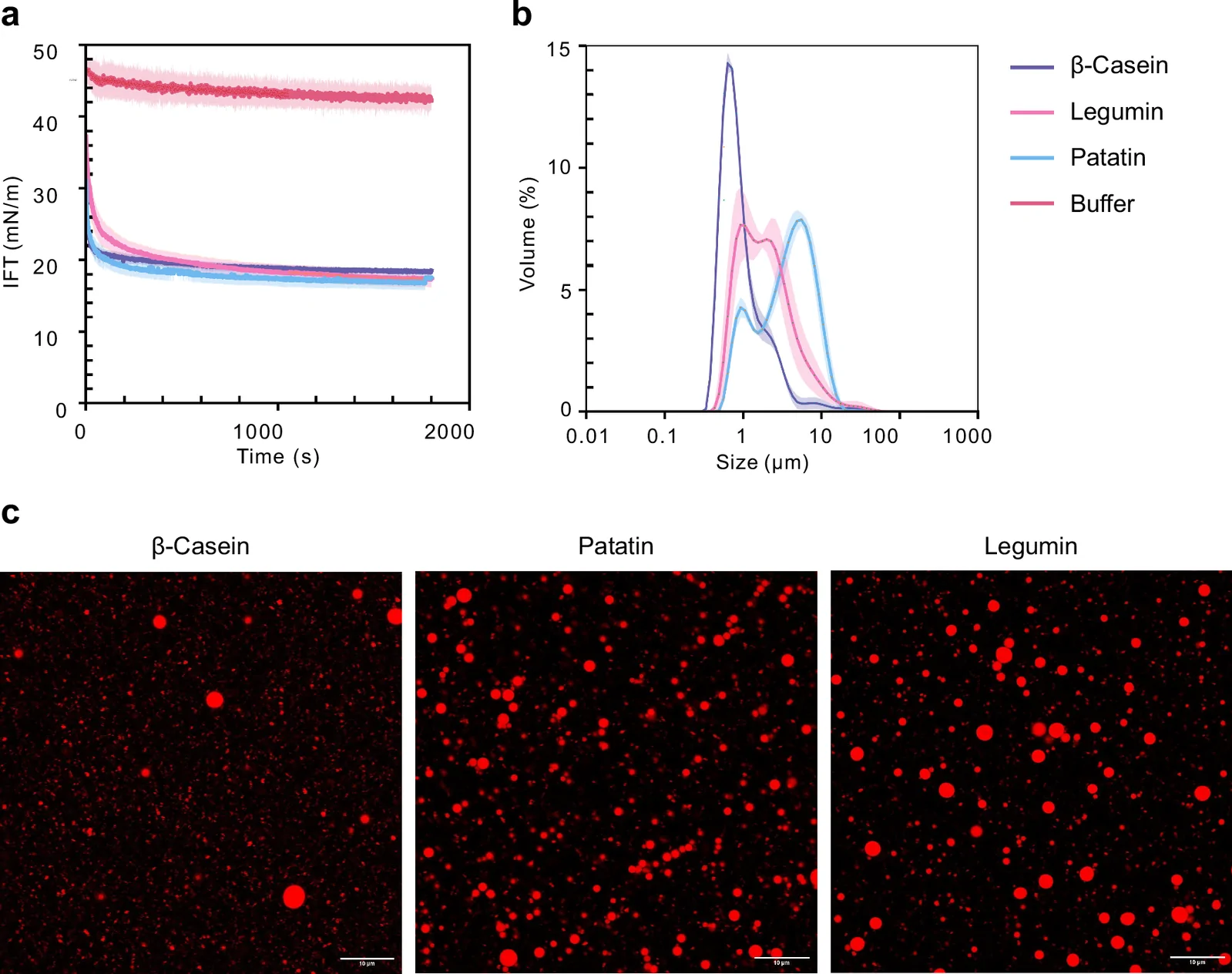
Experimental validation of disordered diblock-like plant proteins acting as optimal surfactants. **a** Interfacial tension (IFT) evolution in the presence of 0.01 wt.% legumin B or patatin as compared to of *β*-casein, as a function of time at the *n*-tetradecane-4- (2-hydroxyethyl) piperazine-1-ethane-sulfonic acid (HEPES) buffer interface at pH 7- and 20-mM NaCl concentration. Legumin and patatin were chemically disordered by addition of 8 M urea and 10 mM dithiothreitol (DTT). The showing interfacial tension (IFT) for the bare interface containing DTT and Urea is also shown as a control (buffer without any protein) is 45 mN/m in agreement with previous literature^51^. All three proteins show a similar ability to decrease IFT with time. **b** Droplet size distribution of freshly prepared oil droplets stabilized *β*-casein, legumin B or patatin ( + urea and DTT) at pH 7 and 20 mM NaCl concentration. Legumin B- and patatin-stabilised droplets resemble droplet size distribution of *β*-casein-stabilised droplets with some variations; latter has the smallest and narrowest droplet size distribution among the tested samples. **c** Fluorescent images of the corresponding oil droplets stabilized by *β*-casein, patatin, and legumin B with oil fluorescing due to the added Nile Red, coloured in red (Excitation: 520 nm) and protein due to Fast green FCF, coloured green (Excitation: 633 nm). Note proteins fall below the resolution of the CLSM and therefore green is not visible in the images. Scale bars represent 10 um in the images. Solid line data shown in (**a**, **b**) are means of three independent readings on triplicate samples (*n* = 3 × 3), while the shaded region represents standard deviations.

We sought to experimentally validate if patatin and legumin B, predicted as diblock-like, act as good polymeric oil-in-water (O/W) emulsifiers, like *β*-casein. To arrive at a disordered structure compatible with the SCFT curves (Fig. 4e–g), legumin B and patatin were chemically disordered by addition of 8 M urea and 10 mM dithiothreitol (DTT) (protein profile shown in Suppl. Fig. 11). Both proteins decreased the interfacial tension, similarly, reaching 18.5 mN m^−^^1^ after *ca*. 1500 s, similar to that of *β*-casein (Fig. 5a). In the initial stages of adsorption, *β*-casein and patatin showed a faster decrease in tension compared to legumin B, consistent with the SCFT volume fraction profiles shown in Fig. 4f. Since the final interfacial tension (IFT) values were the same, the attached structures of these two plant proteins arrange into a similar quasi-equilibrium attached state as that of *β*-casein.

O/W emulsions were prepared and compared against those stabilised by *β*-casein (Fig. 5b). While legumin B and patatin yielded droplet distributions ranging in size from 0.5 to 50 µm, similar to those with *β*-casein (Fig. 5c), the latter showed the narrowest and a monomodal droplet size distribution. Aggregation of the droplets stabilized with plant proteins likely stems from insufficient repulsive potential at close separations ( < 5 nm), as predicted by the SCFT calculations and our ML-based ranking (Fig. 4g) which shows that, although similar, the two plant proteins are not exactly equivalent to *β*-casein. We also tested non-diblock plant proteins, such as 11S sunflower seed globulin with a low *D(Q)* score 0.27. Notably, sunflower 11S could not form stable emulsions compared to the diblock-like proteins. The 11S sunflower protein adsorbs in a train-like configuration (see Supplementary Fig. 12) explaining why emulsion droplet-stabilised by 11S sunflower protein are likely to coalesce, validating the utility of our approach (see Supplementary Fig. 13).

In synthetic diblock surfactants, adsorption and stabilisation are influenced by block lengths, block asymmetry, and adsorption energy. Colloid chemists have long used these parameters to design diblock polymeric surfactants^3,14,41,42^. In our work, the 14-dimensional space provides an analogous design space for synthesizing or selecting proteins that will behave as diblock-like surfactants to replace synthetic surfactants (Fig. 4a). Although our discovery pipeline is focussed on plant proteins, our work classifies the 20 common AAs broadly into 6 types ((positive, negative, hydrophobic, polar and Histidine and Phosphoserine) and uses Flory-Huggins parameter to control adsorption, the pipeline is therefore extendable to a natural, or any synthesized proteins comprising of these 20 AAs to predict their surfactant behaviour considering these proteins as coil-like disordered polymers. The assumption that the proteins are flexible and coil-like is a limitation in SCFT model used in this pipeline. While many proteins, including plant proteins, can undergo partial unfolding inherently due to processing or upon adsorption at interfaces, the findings presented here are most directly applicable to adsorption problems in which configurational flexibility plays a dominant role, consistent with the chemical denaturation employed in this study. Incorporating protein-specific structural rigidity and folding constraints upon adsorption represents a necessary future direction of this discovery pipeline and is beyond the capability of SCFT modelling. Such structural extensions would further expand the applicability of the pipeline to various degrees of unfolding of proteins in industrially relevant conditions.

When dealing with natural proteins, the presence of secondary minor proteins is important to consider. In this study, we have focused on the major proteins present in each of the source tested to minimize the effects of smaller protein fractions. Purified *ß*-casein was purchased, Legumin was purified from pea, while patatin is the major protein in potato^46^ and 11S globulin^47^ is the major protein in sunflower (Suppl. Fig. 11). However, due to the nature of such materials, it is not realistic to eliminate the influence of minor proteins unless targeted production methods are used. Also, we acknowledge that validation using a range of highly ranked protein is a necessary endeavour which can be carried out in future by expressing these proteins in high-throughput experimental work. To address both these shortcomings, research needs to focus on targeted production of proteins of interest in sufficient quantity for experimentation via precision-fermentation or other biotechnological approaches.

## Conclusions

This work establishes a statistical mechanics-embedded, discovery pipeline for identifying plant-based surfactants through adsorbed configuration fingerprinting. By integrating self-consistent field theory with machine learning, we reveal that block-level AA features determine adsorption configuration, unlike protein sequence-level features, challenging conventional thinking. We show the quantitative importance of seven domain-specific features per block for protein adsorption and adopted configuration on surface. The robust methodology presented herein, facilitates a high-throughput screening of millions of proteins to discover protein-based surfactants, advancing us beyond heuristic approaches. We envisage that this data-driven discovery pipeline, underpinned by statistical thermodynamics insights and constructed entirely from open-source protein repository and codes, should find widespread applications in solving other forms of adsorption and wetting problems in colloid science, beyond those aimed at identifying optimal protein-based polymeric surfactants.

## Methods

### Materials

*N*-tetradecane, sunflower oil, *β*-Casein (98% purity), NuPAGE sodium dodecyl sulphate polyacrylamide gel electrophoresis (SDS-PAGE) gels, analytical grade Urea and Dithiothreitol (DTT) and 4- (2-hydroxyethyl) piperazine-1-ethane-sulfonic acid (HEPES) were obtained from ThermoFischer (Altrincham, UK). Legumin B was extracted in laboratory from pea flour, and it had a purity of 85 wt%^34,46^. Commercial potato protein was obtained from Sosa (Barcelona, Spain) was used as patatin protein (protein content> 85%)^47^. Sunflower protein concentrate (55% purity) from Suntein^®^ was used as 11S globulin. Double deionized water was obtained using Millipore^®^ and 20 mM HEPES buffer was used as a solvent.

### Modelling and Machine Learning

#### Protein sequence preparation

The protein sequences selected from UniProt database^9^ carry signal peptides in the primary sequence. Therefore, to remove the cellular localization signal peptides, SignalP program, an open-source tool^48^ was used on the selected proteins in FASTA format, and the signalling peptides were removed before performing SCFT.

#### Selection of sequences in the discovery pipeline

The first step of the discovery pipeline was to generate a plant protein database. We developed a five-step process to sample plant protein sequences (Fig. 1): i) multicellular plant proteins (Gunneridae taxonomy) allowed selection of flowering plants from 250 million proteins in PDB repository using UniProt (https://www.uniprot.org/)^9^, followed by further filtering by ii) sequence length (100-2000 amino acids (AAs)) to arrive at 10 million sequences, and only proteins of iii) manual annotation quality that had been experimentally reviewed were selected. Given the large number of sequences (25,000), we further filtered these top candidates to ones having iv) iso-electric point below pH 6.5, to be relevant for most biological^49^ and industrial applications^50^, and these v) 1950 sequences were selected for SCFT calculations based on their size, calculated hydrophobicity and calculated isoelectric point using BioPython^51^.

These 1950 plant protein sequence candidates were modelled as linear chains, with their AA encoded (Fig. 1) as hydrophobic, hydrophilic, charged residues and Histidine (due to its unique pKa)^33,34^ and computed using multiple iterative rounds of SCFT calculations.

#### SCFT calculations

Self-consistent field theory (SCFT) is a numerical tool in statistical mechanics used to predict adsorption, density profiles and interaction parameters of coil-like disordered polymers in the presence of solvent, and electrolytes at equilibrium^26,33,34,52^. The principles of the SCFT, a Density Functional type Theory (DFT), was used here by applying the Scheutjens-Fleer formulation of the theory to protein-like polymers^53^. Briefly, in SCF calculations, the interactions are felt by any monomer unit (AA in our case) with its neighbouring residues, solvent molecules, or ions, which are introduced as a set of effective fields at each position. For monomer AA residues of type *α*, this effective field at position **r** can be expressed as

3 $${\psi }^{\alpha }({{{\bf{r}}}})={q}^{\alpha }{\psi }_{el}({{{\bf{r}}}})\,+\,{\sum}_{i}{\sum}_{\beta \ne \alpha }{\chi }_{\alpha \beta }({\phi }_{i}^{\beta }({{{\bf{r}}}})-{\varPhi }_{i}^{\beta })\,+\,{\psi }_{h}({{{\bf{r}}}})$$

where, the summation *i* is taken over all molecular species present in the system (proteins, solvent, ions, etc). The first term on the right-hand side of the above equation accounts for the long-range electrostatic interactions between the residues, where *q*^*α*^ is the charge of the monomers of type *α*, and *ψ*_*el*_(**r**) the electric potential at position **r**, as determined by the Poisson’s equation, shown below:

4 $${\nabla }^{2}{\psi }_{el}({{{\bf{r}}}})=\frac{-\left({\sum}_{i}{\sum}_{\alpha }{q}^{\alpha }{\phi }_{i}^{\alpha }({{{\bf{r}}}})\right)}{{\varepsilon }_{0}{\varepsilon }_{r}}$$

The second term in the Eq. 3 accounts for the short-range interactions, such as hydrophobic, hydrogen bonding, or any other nearest neighbour interactions between two different sets of monomers. The overall strength of such interactions is characterised by a set of Flory-Huggins parameters {*χ*_*αβ*_}. Typically, the values of these parameters are of the order of a few *k*_B_*T*, where *k*_B_ is the Boltzmann constant and *T* the temperature. The field due to such short interactions, felt by a monomer of type *α* at position **r**, will of course depend on the set of densities {*φ*_*i*_^*β*^(**r**)} of all other species *β*, belonging to all the different chains *i*, around that position. The final term in Eq. 3 is a hard-core potential, enforcing the incompressibility of the system, which is simply expressed as

5 $${\sum}_{i}{\sum}_{\alpha }{\phi }_{i}^{\alpha }({{{\bf{r}}}})={\sum}_{i}{\sum}_{\alpha }{\varPhi }_{i}^{\alpha }$$

Through an appropriate statistical mechanics averaging process, usually required to be carried out numerically, the variation of volume fractions {${\phi }_{i}^{\alpha }$ (**r**)} can be evaluated^53,54^. The resulting volume fractions are then used to calculate a new set of fields using Eq. 3. The processes are repeated many times until convergence is obtained.

In this study, we apply SCF calculations to predict adsorption profiles of plant proteins derived from UniProt along with bovine *β*-casein. The dispersed system is simulated with SCF across a gap between two planar surfaces whose separation distances are separated as individual layers each consisting of a regular cubic lattice spacing. Each monomer of the protein, solvent molecule and ion occupy a fixed unit cell space of size ~0.3 nm (nominal size of a peptide bond). The density profile distribution for each species, with the variations perpendicular to the surface across the gap, which yield the lowest free energy is calculated. A detailed explanation of principles of SCF as well as the underlying equations are provided in earlier works^26,33,34,52^. SCF calculations were performed at pH 7.0 and NaCl volume fraction of 0.0001 (equivalent to 1 mM). The Flory-Huggins parameter for different monomer types, used in the SCF calculations to determine density profiles, are provided in the Suppl. Table 3. The obtained SCF calculation data were analysed, visualized and organized using Python.

#### Machine Learning Methods

All analysis were performed using Google Colab IDE environment using python. Machine Learning (ML) clustering functions such as DBSCAN (Density Based Spatial Clustering of Applications with Noise), K-means clustering, Kernel K-means were used. These are unsupervised clustering methods used to find natural clusters (similarity) of data using a set of features. We also used Random Forest classifier (RFC) as an interpretation tool. Logistic regression and regression models were used to classify attached and free blocks. A classifier in ML classifies data into two or more classes based on training on known classified classes. Random Forest is a decision-tree based classifier whereas logistic regression classifier, classifies an input between binary categorical choices, based on a sigmoid function. Gradient Boosting is an ensemble model, which uses multiple decision trees and a sequential training method unlike. Dimensionality reduction techniques Uniform Manifold Approximation and Projection (UMAP) and t-stochastic neighbour embedding (t-sne)^55^ were used. These methods are employed when more than 3 or 4 features (parameters) are used, to enable visualization and computing in 2-d from multi-dimensional feature space. All these frameworks were all part of standard SciKit learn^56^. Statistical analysis was performed using python package Pingouin, which provides a streamlined way to perform statistical analysis^57^. Numerical analysis used NumPy python package which provides capabilities to perform numerical operations within python^58^. Data storage and excel creation were handled using pandas python package, which can read and write, and retrieve Microsoft excel data. Visualizations used matplotlib^59^, and Scienceplots package, which enables advanced plotting capabilities within python environment appropriate for scientific work. ProtTrans protein language model as benchmark for clustering analysis. ProtTrans converts protein AA sequences into numerical array called embeddings^43^. These embeddings contain positional and physico-chemical properties of AAs, which were extracted using ProtTrans opensource code available on the GitHub repository.

#### Protein-level feature extraction for K-means clustering

The AA were coded as hydrophobic, hydrophilic uncharged, positive, negative charged and histidine across all AAs. The AA belonging to each category is shown in the Supplementary Table 2. Python 3.12, run on Google Colab was used to categorize each of the AAs into numbers 1 through to 5. 1 being hydrophilic, 2 hydrophobic, 3 negative and 4 positive charged and 5 histidine. Then the different types were counted such that total hydrophilic is the count of both uncharged and charged hydrophilic AAs as well as Histidine (sum of types 1,3,4 and 5) whereas total charged was summation of AAs of type 3 and 4. Total hydrophobic AA is simply the total type 2 in a chain and finally the total length is simply the length of the AAs in the protein. Also, the number of times AA of type 2 switches from or to another type is counted and double counting is avoided by only counting either switching to or switching from of any consecutive type 2 AA.

#### Adsorbed configuration features

After SCFT calculations, the adsorbed configuration features were extracted also using Python on Colab environment. An AA at 0.3 nm distance is at the surface in the strict sense. However, slight variations from the 0.3 nm strict cut-off might occur, but still the AA could be considered ‘attached’ on the surface. Therefore a 20% deviation from the hard cut-off of 0.3 nm (0.36 nm) was used as a threshold of 0.36 nm for classifying each AA as attached or free. An AA was considered attached if its distance value ≤ 0.36 nm and was considered free if the distance value was > 0.36 nm. The number of points along the chain in these two categories was calculated as attached and free lengths, respectively. Similarly, if the first point (N terminal) was found to be above 0.36 nm and at least 10 AAs on a 10 AA rolling average were above 0.36 nm distance from the surface, then a head free length was calculated. In the same way the last AA (C-terminal) was checked and if the 10 preceding AAs were on a rolling 10 AA average at a distance greater than 0.36 nm then a tail free length were calculated. The two sets of features were merged and the correlation plots and heatmap were constructed using Python on Google colab environment using Matplotlib Science plots framework.

#### K-means clustering using features

The two sets of features were used as input to unsupervised clustering algorithm, kernel *K*-means clustering. *K*-means clustering is an unsupervised clustering framework that groups points by iteratively assigning them to the nearest average of a group of points and regrouping. A rbf kernel (radial basis function kernel), which measures how close two points are in Euclidean coordinates was applied to the standardized data and *K*-means was applied to identify three clusters. Three clusters were chosen based on elbow plot, showing inertia decreases sharply up to three clusters and then further clusters show weaker decrease in inertia. Silhouette score was calculated using the scikit learn metrics framework.

#### ProtTrans embedding based clustering

For each protein in the 1858 proteins SCFT model calculations, the corresponding ProtTrans embeddings were extracted, yielding high-dimensional feature per monomer representation of each protein. ProtTrans is a Protein Language Model, which is specifically created to decipher the AA sequence properties of proteins and is trained on millions of protein sequences. These advanced models serve as benchmark ML models for proteins. Therefore, the embeddings, which are the numerical representations of the AA sequence properties of ProtTrans were extracted for each of the proteins. These embeddings served as training inputs to a neural network designed to predict the full SCFT curve. Specifically, a Long Short-Term Memory (LSTM) network to map the ProtTrans features to the associated SCFT curve was trained. This was carried out using pytorch 2.9.1 (https://pytorch.org). After training, the activations from the final hidden layer of the LSTM were extracted as a lower-dimensional latent representation of each protein. To examine whether this learned latent space captured any existing knowledge structure, the representations were visualised using a t-SNE and annotated using the binary configuration feature extraction method described in the DBSCAN clustering section. The results were visualized using t-stochastic neighbour embedding (t-sne) embedding, which is a dimension reduction technique to visualize high dimensional data in 2- or 3-dimensions using probability measure of similar points being nearby.

#### Adsorption configuration feature extraction and DBSCAN clustering

To apply clustering, four binary (0 or 1) features were created for the proteins based on their SCFT adsorbed configuration. These were ‘Tall head,’ ‘Tall tail’, ‘Tall middle’ and ‘Multiple tall’ sections. To avoid noisy sections influencing the features, the distance profile was averaged using a 10AA rolling average before feature extraction. To apply the binary tall section feature, three distance classes were created to approximately capture the shape and overall block-shape of the adsorbed protein configuration. The first criteria were AAs at an average distance of < 0.36 nm is considered ‘attached’ and AAs above 1.2 nm are considered ‘free.’ 0.36 nm represents surface + 20% deviation as mentioned in earlier section. In contrary, larger than 1.2 nm was chosen as criteria for free as it represents distance away from the surface, where the AA can be considered not influenced by the presence of the surface. Thus, in SCFT calculations, 1.2 nm represents 4 lattice units away from surface. AAs between 0.36 nm and 1.2 nm average distance were either considered attached or free depending on the sections neighbouring them on either side. For instance, a section was only considered tall, if it extended above 1.2 nm from the surface. The criteria were applied to SCFT distance curves. The first AA (N-terminal) all the way up to at least 10 AAs were checked if they had an average distance value of at least 1.2 nm - if this criterion was true, then ‘head tall’ feature was ‘1’, else it was considered false and ‘0’ was assigned for this feature (Supplementary Table 1). Then the presence of ‘multiple tall’ sections was checked if there were more than 1 tall section based on at least 10 AA along the chain at an average height > 1.2 nm, then that counted as ‘0’ or ‘1’ based on if they did not exist or if they existed, respectively.

#### Unsupervised clustering using DBSCAN

The binary features were used to cluster the SCF curves using density based unsupervised clustering algorithm Density Based, DBSCAN. DBSCAN clusters points that are closely packed (density-based) and marks isolated points as noise. The DBSCAN clusters were dimension reduced using Uniform Manifold Approximation and Projection (UMAP), which is a topological dimensionality reduction technique, which represents higher dimensional space into a 2-d graphical format. Also, the clustering effectiveness was checked using Silhouette score (Scikit learn) which goes from -1 for poor clustering to +1 for perfectly separable clustering. The cluster labels and proteins in each cluster were identified, and the distance profiles were verified for cluster similarity.

#### SCFT and adsorption configuration-based diblock scores

For an AA at distance d from the surface in the adsorption configuration of the protein from SCFT, we created a hydrophobicity scale based on its distance from the surface. We gave an AA a hydrophobicity score p=1 if d≤0.36 nm, p=0 if d>1.2 nm and p=0.5 otherwise. The hydrophilicity score q was assigned as q=1−p. Then the average hydrophilicity and hydrophobicity over a block h or t was computed to obtain γ and η. The computation was conducted over all possible segmentations of the sequence and repeated on the reversed sequence to incorporate cases where *h* is hydrophobic, and *t* is hydrophilic.

#### Block-level feature extraction

From the unsupervised clustering, 787 diblock like proteins are identified (Fig S3), which are divided into one attached and free block per protein. The first point below 0.36 nm was identified along the protein chain as the cut-off point. The index was used to split the AA sequence into attached block and free block sequences. Seven domain specific features were extracted from each block. The block length, Fraction of polar (Sum of type 1 and type 5 AA as fraction of total block length), Fraction charged (Sum of Type 3 and 4 AA as a fraction of total AA in the block), Fraction hydrophobic (Type 2 as a function of block length), Hydrophobic vs polar, Hydrophobic vs charged, and Polar vs charged AAs for each block. So, each block had six features, and every diblock protein comprised of two sets of six features (14 total features). The features were computed using python and statistical analysis were performed. First Welch’s independent t-test was performed, subsequently p-test was conducted using python pingouin statistics package. The distributional difference in AA between attached and free blocks were visualized using Violin plots. Violin plots show the distribution of block-level features for attached and free blocks across diblock-like proteins. The width of each violin represents the probability density of the data, while the central marker indicates the median value. Differences in the shape and median of the violins indicate systematic differences between attached and free blocks.

#### Random forest classification for block-level feature importance

The block-level features of diblock proteins were used to train an ensemble method, a random forest classifier (RFC), as a interpretation tool to extract the importance of features in distinguishing attached and free blocks. We used SCF derived features and labels to train and test the model and therefore can be considered circular, however, we only use RFC for interpretation.

For simplicity, no hyper parameters were tuned and the default random forest classifier training parameters provided through SciKit learn were used. Grouped K-fold cross validation was applied, where the data was split into train and test set and during split, both attached and free blocks of the same protein were grouped together as either training or test data for each fold. A 5-fold validation was performed, where the total data is split as grouped fold 5 times and each time the classifier was run, and the performance and the average accuracy metrics were computed over the 5 folds and reported. Specifically, we used a classifier feature importance (SciKit learn), which ranks importance based on how often the classifier used the specific feature to predict whether a block is attached or free. RFC enabled extracting how important each of the feature were to train and distinguish attached and free blocks, which is referred to as feature importance. The feature importance was calculated for each of the grouped 5-fold cross validation results and the average of the 5-fold validation was reported.

The feature importance provides a fractional importance for each feature used, add up to 1. Therefore, the importance of our 7 features was visualized as a ranked-feature importance bar chart or so-called waterfall plot.

#### Euclidean distance metric for plant protein ranking

The two sets of seven features from each blocks for the diblock protein were used to compute a 14-dimensional distance metric to assess protein similarity and compare them to *β*-casein. We used feature importance obtained from the random forest classifier training as weights for the 14-dimensional feature space. Using the feature importance as weights and *β*-casein as reference point, we computed distance of each protein from *β*-casein. The 14-dimensional space was diminished to a 2-dimensional representation using PCA and the linear Euclidean distance was computed and used to rank the proteins.

### Experiments

#### Chemical denaturation of plant proteins

Protein solutions of appropriate concentrations were prepared using HEPES buffer at pH 7.0. Plant proteins were chemically disordered using an 8 M urea and 15 mM DTT solution^60^. All protein dispersions were prepared to standardized protein content based on their purity to enable comparison between their interfacial and emulsion properties. In case of sunflower, lower purity was compensated by having the same protein content in emulsions to be tested against legumin and patatin.

To qualitatively measure protein composition, sodium dodecyl sulphate-polyacrylamide gel electrophoresis (SDS-PAGE) was used. Specifically, about 1 mg/mL protein solution was prepared by dispersing the proteins in HEPES buffer at pH 7.0. A pre-stained Protein Standard Novex^TM^ Sharp Pre-Stained Protein Standard comprising 4 kDa up to 200 kDa was used as a reference. The gels were run using MEPS running buffer for about 35 min until the protein bands were well separated. After staining, the gels were de-stained using DI water for 1 h then scanned using Bio-Rad gel scanner. The images of the gels were used further without any modification to identify molecular weight bands of the proteins.

#### Interfacial tension

Dynamic interfacial tension measurements were performed using an OCA 25 (Dataphysics Instruments, Germany) drop shape tensiometer, with *n*-tetradecane as the hydrophobic phase and HEPES buffer or protein solution in buffer used as the aqueous phase. For all measurements, a protein solution of concentration = 0.01 wt.% was prepared by dispersing the protein in HEPES buffer at pH 7.0. An 18 µL droplet was formed at the edge of the needle and temperature was maintained at 20 °C using a circulating water bath. Tension measurements were performed for 30 min.

#### Emulsion characterisation

To investigate the ability of proteins to stabilize emulsions, a 10 wt.% sunflower O/W emulsion was prepared. Briefly, about 5 mg/mL protein dispersion was prepared by mixing in HEPES buffer for 2 h to facilitate protein dissolution. The protein dispersion was stirred using high speed rotor stator mixer (Ultraturrax, IKA, Oxford, UK) for 1 min at 6000 rpm and while the oil phase was added slowly and the mixture was homogenized at 10,000 rpm for 1 min. The coarse emulsions were further homogenized using the custom-made Leeds Jet Homogenizer (University of Leeds, UK) at 300 bar pressure using three consecutive passes. To assess the emulsification ability, droplet size of formed emulsions was measured using laser diffraction in a Malvern Mastersizer 3000 (Malvern Panalytica, Malvern, UK) and confocal laser scanning microscopy (Nile Red fluorescing droplets, Excitation: 520 nm, Fast Green FCF fluorescing proteins, Excitation: 633 nm). A refractive index of 1.4 was used for sunflower oil and refractive index of water was set to 1.33 in Mastersizer. To measure droplet size of individual droplets and to disrupt any weakly flocculated aggregates, emulsions were treated with 1 wt.% sodium dodecyl sulphate (SDS) prior to size measurement. All measurements were conducted in triplicate.

## Supplementary information

Supplementary Material

ML- Reporting-Summary

## Data Availability

All data are available in the Article or its Supplementary Information. Supplementary data relevant for this publication are provided with this paper in the following link: https://doi.org/10.5518/1824.

## Code availability

The code for the data driven discovery pipeline used in this study are freely available for download from the online repository: https://github.com/thazlehurst/ProSurf. The code is also archived in Zenodo^63^.
