# Supplementary material for: Data-driven pipeline enables discovery of plant protein surfactants
Source: Commun Chem. 2026 Sep 3;9:281. doi: 10.1038/s42004-026-02173-6 (PMC13542254; doi:10.1038/s42004-026-02173-6)
Supplement: Supplementary file 2 [file 42004_2026_2173_MOESM1_ESM.docx]

***Supplementary Information***

**Data-driven pipeline enables discovery of plant protein surfactants**

Simha Sridharan^1****^, Rammile Ettelaie^1***^, Rik Sarkar^2**^, Maryam Afzali Haji Dela^1,3^, Brent S. Murray^1,3^, Thomas A. Hazlehurst^1,3^, Nicholas J. Watson^1,3^, Anwesha Sarkar^1,3*^

^1^ Food Colloids and Bioprocessing Group, School of Food Science and Nutrition, University of Leeds, Leeds, UK

^2^ School of Informatics, University of Edinburgh, UK

^3^ National Alternative Protein Innovation Centre (NAPIC), Leeds, UK

Corresponding authors

*** Simha Sridharan: [L.Sridharan@leeds.ac.uk](mailto:L.Sridharan@leeds.ac.uk)

** Rammile Ettelaie: [R.Ettelaie@leeds.ac.uk](mailto:R.Ettelaie@leeds.ac.uk)

** Rik Sarkar: [rsarkar@inf.ed.ac.uk](mailto:rsarkar@inf.ed.ac.uk)

* Anwesha Sarkar: [A.Sarkar@leeds.ac.uk](mailto:A.Sarkar@leeds.ac.uk)

**Fig. 1| Exemplar adsorption configurations of a subset from 1,858 plant proteins.** Exemplar adsorption configuration curves of proteins obtained from SCFT showing diverse shapes of protein configuration, such as diblock-like, loop-like *etc.* with number of amino acids (AA) along the X-axis and the distance away from a hydrophobic surface along the Y-axis. **(a)** Putative FBD associated Fbox protein, length 468 AAs, **(b)** Glucose6phosphate isomerase cytosolic 2, length 569 AAs. **(c)** Germin-like protein subfamily 1 member 8 Arabidopsis thaliana, length 198 Aas, **(d)** Chlorophyllase 1 Chloroplastic Citrus sinensis, length 329 AAs, **(e)** Alpha amylase 1 Arabidopsis thaliana, length 399 AAs, **(f)** Scarecrow like protein 15 Arabidopsis, length 486 Aas **(g)** Two pore calcium channel protein 1B Nicotiana tabacum, length 735 AAs. **(h)** Bidirectional sugar transporter SWEET17 Arabidopsis thaliana, length 241 AAs, **(i)** gamma cadinene synthase Cucumis melo, length 571 AAs.

**Fig. 2| Comparison of AA properties of converged versus unconverged proteins. a.** Fraction charged AA of unconverged proteins against converged proteins. **b.**  Fraction hydrophobic AA of unconverged proteins against converged proteins. **c.** Distribution of length of proteins of unconverged proteins against converged proteins. **d.** Fraction polar AA of unconverged proteins against converged proteins. Number of converged proteins n= 1858, Number of unconverged proteins n**=**92

**Fig. 3|** **Diblock-like** **configuration of *β*-casein upon adsorption**. **a.** Schematic of *β*-casein as a linear chain of positive, negative, hydrophilic, hydrophobic amino acids, with additional separate groups for each of histidine and phosphoserine residues. Histidine is modelled separately due to its specific distinct pK_a_ and phosphoserine is a special amino acid, possessing two pK_a_, and thus also modelled separately. **b.** The average distance of each monomer of *β*-casein relative to a hydrophobic surface, with amino acids labelled consecutively as 1, 2, 3, …., starting from the N-terminus. The adsorbed configuration of *β*-casein along the chain of monomers of the protein. The shape of the average distance curve resembles a ‘di-block’ like structure with a free, extended section at the N-terminal (termed here as head) and a continuous ‘attached’ section, lying almost flat on the surface, that goes all the way to the end C-terminal (termed here as tail). Specifically, a distance value of ~ 0.3 nm (one monomer size) indicates that the AA is residing on the surface most of the time. On the other hand, higher values show less likelihood that the residue will be found on the surface, *i.e.*, the blocks tend to be ‘free’ and part of a dangling section protruding away from the surface into the solution. **c.** Interaction potential in units of *k*_B_*T* (where *k*_B_ is Boltzmann constant and *T* is temperature in Kelvin (K)) induced by *β*-casein between two hydrophobic spherical surfaces covered with this protein, plotted as a function of separation distance between the two spheres of diameter 1 µm calculated using Derjaguin’s approximation. **d.** Volume fraction of *β*-casein as a function of distance from the hydrophobic surface shows a high density close to the surface, indicating its affinity to attach to a hydrophobic surface, followed by a sharp decrease at distances further from the surface.

**Fig. 4|** **Binary feature selection from SCFT.** Four binary features have been used to describe the presence of tall segments at different positions along the AA chain upon adsorption. **a.** The curve only has one major tall peak at the N-terminal called head; therefore, the binary feature is 1. The rest of the positions do not contain any tall segments and are 0. **b.** The curve shows multiple peaks at the beginning, at the end and in the middle. Therefore, it has tall head, tall tail, tall middle and multiple tall segments. All four binary features are 1.

**Fig. 5| Exemplar adsorption configurations of plant proteins from the DBSCAN clusters.** Exemplar distance curves of proteins based on their DBSCAN cluster with number of amino acids (AA) along the x-axis and the distance away from surface along the y-axis. **(a)** Example cluster 0: Protein BRI15 Enhanced 1 Arabidopsis thaliana OX3702 GNBEN1 PE2, length 364 AAs, **(b)** Example cluster 1: Cytochrome c oxidase subunit 2 mitochondrial Fragment Vigna ungu, length 376 AAs, **(c)** Example cluster 2: Nascent polypeptide associated complex subunit alphalike protein 3, length 204 AAs, **(d)** Example cluster 3: Plastid division protein CDP1 chloroplastic Arabidopsis thaliana O, length 819 AAs, **(e)** Example cluster 4: Autophagyrelated protein 3 OS Arabidopsis thaliana, length 313 AAs, **(f)** Example cluster 5: SKP1like protein 7 Arabidopsis thaliana, length 125 AAs, **(g)** Example cluster 6: Patatinlike protein 6 Arabidopsis thaliana, length 499 AAs, **(h)** Example cluster 7: Kinase interacting family protein Arabidopsis thaliana, length 297 AAs, **(i)** Example cluster 8: Gtype lectin S receptor like serinethr Arabidopsis thaliana, length 800 AAs.

**Fig. 6| Number of clusters and their adsorbed configurations.** DBSCAN cluster results based on binary features applied to 1,858 proteins. **a.** Bar chart showing the number of proteins identified in each of the 9 different clusters in DBSCAN. **b.** Average adsorbed configuration for each of the 9 clusters identified by DBSCAN, shown here as normalized height and normalized length to avoid variations in length and heights of proteins in these different clusters. **c.** Illustration of attached and free block segmentation for further analysis.

**Fig. 7| SCF distance curves representing examples of diblock-like plant proteins ranked from *β*-casein.** **a.** Rank 3, Thaumatinlike protein 1b Fragment OSMalus domestica OX3750 PE2 S, length 212 AAs, **b.** Rank 23, Nascent polypeptideassociated complex subunit alphalike protein 1 OS, length 203 AAs, **c.** Rank 76, ATP synthase subunit d mitochondrial OSArabidopsis thaliana OX3702, length 168 AAs, **d.** Rank 163, 1aminocyclopropane1carboxylate oxidase OSPrunus mume OX102107 GN. **e.** Rank 220, Eukaryotic translation initiation factor 2 subunit alpha OSArabidopsis, length 344 AAs, **f.** Rank 379, Sadenosylmethionine synthase 1 OSSolanum tuberosum OX4113 GNMETK1, length 393 AAs, **g.** Rank 447, Uridinecytidine kinase UKL1 chloroplastic OSArabidopsis thaliana OX, length 486 AAs, **h.** Rank 615, Putative DNAdirected RNA polymerase subunit alphalike 1 OSPelargoni, length 574 AAs.

**Fig. 8|** **Comparison of block-level features in the diblock-like plant proteins with diblock score (D(Q)) ~ 0.5. a.** Bar chart showing the average values for the six features for attached and free blocks of the 787 diblock like proteins. The error bars represent standard deviation. **b.** T-test bar graph showing the difference between the values of each feature between attached and free blocks. All six features have high t-values, indicating significant difference between the block-level features. **c.** Bar chart showing the -log P value of the six features, showing that all the six features show very small p-value difference between attached and free blocks. Together, t-value and p-values for the features between attached and free blocks indicate significant statistical difference in compositional features of the blocks within these n= 787 diblock-like plant proteins.

**Fig. 9| Random Forest classifiers applied to free and attached blocks in diblock-like plant proteins with diblock score (D(Q)) ~ 0.5.** Violin plots showing distribution of features at the block-level in adsorbed and free blocks used in random forest classifier for 787 proteins. **a.** Violin plot for fraction hydrophobic AA. **b.** Violin plots showing distribution of charged vs hydrophobic AA. **c.** Violin plots showing block length distribution. **d.** Violin plot of fraction charged AA in attached and free blocks. **e.** Violin plots showing fraction polar in attached and free blocks. **f.** Violin plot showing polar vs hydrophobic AA in attached and free blocks. **g.** Violin plot showing charged vs polar AA in attached and free blocks. Note: Each violin plot is interpreted within its own panel to compare attached and free blocks for a given feature; comparisons are not made across different features, and differences in axis scaling do not affect the qualitative conclusions. n = 787 attached and free blocks

**Fig. 10| Area under curve (AUC) of Logistic Regression and Gradient Boosting model**. AUC curve discriminating adsorption state of blocks to benchmark against Random Forest. Both alternative classifiers show similar AUC.

**Fig. 11| Chemical composition upon chemical disordering of legumin and patatin.** Sodium dodecyl sulphate polyacrylamide gel electrophoresis (SDS-PAGE) images of the *β*-casein, legumin and patatin proteins used in experimental validation upon chemical disordering of structure using Urea-DTT treatment. *β*-casein has a major band around 30 kDa as expected, since *β*-casein remains disordered regardless of the urea-DTT treatment. Legumin is expected to be around 60 kDa, but due to the chemical treatment, the disulphide (S-S) bond is broken, and the protein chain splits into alpha and beta chains with major bands around 35 and 20 kDa. Patatin is expected to be close to 40 kDa, and unlike legumin, patatin does not contain S-S bonds and so its molecular weight is unaffected by urea treatment and shows the most intense patatin band around 40 kDa, with some other bands around 35-15 kDa.

**Fig. 12| SCFT results of 11S sunflower seed globulins compared to *β*-casein.** **a.** Adsorption configuration of 11S sunflower seed globulins showing a more random train-like configuration unlike deblock-like in *β*-casein. **b.** Volume fraction of sunflower protein and *β*-casein as a function of distance from the surface. Data shows both sunflower protein has > 0.8 volume fraction close to the surface, whereas *β*-casein has a volume fraction of 0.96 at the surface, indicating higher propensity to reside at the surface. **c.** Interaction potential of spherical surfaces (where *k*_B_ is Boltzmann constant and *T* is temperature in Kelvin (K)) induced by *β*-casein or sunflower proteins between two hydrophobic spherical surfaces covered with this protein, plotted as a function of separation distance between the two spheres of diameter 1 µm calculated using Derjaguin’s approximation. *β*-casein has a high repulsive potential at the surface that sharply decreases to 20 x 10^3^ k_B_T at 10 nm separation, whereas sunflower protein starts below 20 x 10^3^ k_B_T and decreases to 0 k_B_T before 10 nm separation, indicating weak repulsive interaction.

**Fig. 13| Experimental validation of disordered non-diblock-like plant proteins failing to act as optimal surfactants. a.** Droplet size distribution of freshly prepared oil droplets stabilized by *β*-casein and 11S sunflower seed globulin (where proteins have been chemically disordered using +urea and DTT) at pH 7.0 and 20 mM NaCl concentration. Sunflower 11S globulin protein-stabilised droplets form larger droplet size distribution with median droplet size of 50 μm unlike *β*-casein, latter showing median droplet size close to 1 μm. **b.** Fluorescent images of the corresponding oil droplets stabilized by sunflower protein showing significant droplet coalescence with oil fluorescing due to added Nile Red for oil droplets (Excitation: 520 nm) and Fast green FCF for protein) (Excitation: 633 nm). **C.** Interfacial tension reduction over time between Hexadecane and HEPES buffer, b-casein and, sunflower protein measured at 20 C. Solid lines of data shown in **a and c** are means of three independent readings on triplicate samples (n = 3 × 3), the standard deviation is presented as shaded region.

**Table 1**| Explanation of four binary features used to group proteins based on their adsorption configurations.

| **Feature** | **Description** | **Present** | **Absent** |
| --- | --- | --- | --- |
| Tall start | N-terminal and minimum 10 AA that follow > 1.2 nm | 1 | 0 |
| Tall end | C-terminal and a minimum 10 AA that precede > 1.2 nm | 1 | 0 |
| Tall middle | 10 or more AA > 1.2 nm along the sequence not including N and C-terminal | 1 | 0 |
| Multiple tall | 2 or more sets of at least 10 AAs > 1.2 nm anywhere along the chain | 1 | 0 |

**Table 2.** Comparison of Different classifier models including Random Forest Classifier used as interpretation tool.

| **Model** | **Average Accuracy** | **Average AUC** |
| --- | --- | --- |
| **Random Forest** | 0.8639 | 0.9411 |
| **Logistic Regression** | 0.8378 | 0.9156 |
| **Gradient Boosting** | 0.8639 | 0.9418 |

**Table 3.** Flory-Huggins interaction parameters for different components used in SCFT calculations.

| **Monomer type** | **0** | **1** | **2** | **3** | **4** | **5** | **6** | **7** |
| --- | --- | --- | --- | --- | --- | --- | --- | --- |
| 0-Solvent | 0 | 0.0 | 2.0 | 0 | 0 | 0 | -1.0 | −1.0 |
| 1- Polar non-charged | 0 | 0 | 2.5 | 0 | 0 | 0 | 0 | 0 |
| 2-Hydrophobic | 2.0 | 0 | 0.0 | 2.5 | 2.5 | 2.5 | 2.5 | 2.5 |
| 3-Positively charged | 0 | 0 | 2.5 | 0 | 0 | 0 | 0 | 0 |
| 4-Negatively charged | 0 | 0 | 2.5 | 0 | 0 | 0 | 0 | 0 |
| 5-Histidine | 0 | 0 | 2.5 | 0 | 0 | 0 | 0 | 0 |
| Special AA (Phosphoserine) | 0 | 0 | 2.5 | 0 | 0 | 0 | 0 | 0 |
| 6-Positive ions | −1.0 | 0 | 2.5 | 0 | 0 | 0 | 0 | 0 |
| 7-Negative ions | −1.0 | 0 | 2.5 | 0 | 0 | 0 | 0 | 0 |
| Surface | 0 | 0 | -2.0 | 0 | 0 | 0 | 0 | 0 |
